# *catena*-Poly[lead(II)-[μ-2,4-diamino-6-(piperidin-1-yl)pyrimidine *N*-oxide-κ^2^ *O*:*O*]di-μ-iodido]

**DOI:** 10.1107/S1600536809020972

**Published:** 2009-06-06

**Authors:** Maryam Ranjbar, S. Heidar Mahmoudi Najafi, Seik Weng Ng

**Affiliations:** a Institute of Chemical Industries, Iranian Research Organization for Science and Technology, PO Box 15815-358, Tehran, Iran; b Department of Chemistry, University of Malaya, 50603 Kuala Lumpur, Malaysia

## Abstract

The *N*-oxide O atom of the minoxidil unit in the 1/1 adduct with lead(II) iodide, [PbI_2_(C_9_H_15_N_5_O)]_*n*_, bridges two Pb^II^ atoms, as do each of the I atoms. The bridging inter­actions give rise to a linear chain motif that propagates along the *a* axis of the ortho­rhom­bic unit cell. The  coordination sphere around the six-coordinate Pb^II^ atom is a distorted ψ-monocapped octa­hedron in which the stereochemically active lone pair caps one of the faces defined by the O and I atoms forming the longer Pb—O or Pb—I bonds. The Pb^II^ atom lies on a mirror plane; the mirror plane is perpendicular to the pyrimidine ring and it bis­ects the piperidine ring. The aromatic ring is disordered about the mirror plane with respect to the 1-nitro­gen and 5-carbon atoms.

## Related literature

For the crystal structure of minoxidil, see: Akama *et al.* (2004 ▶); Martín-Islán *et al.* (2008 ▶).
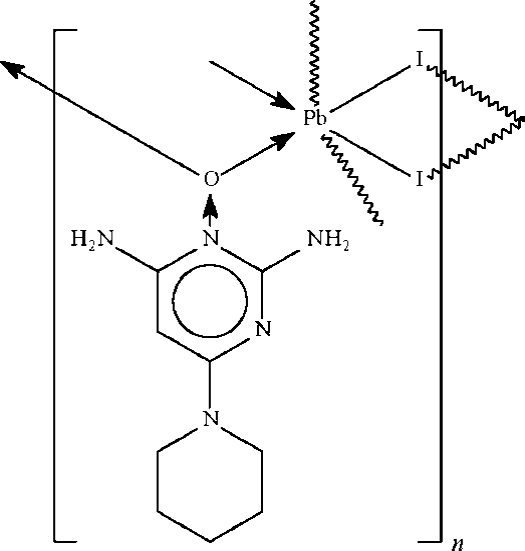


## Experimental

### 

#### Crystal data

[PbI_2_(C_9_H_15_N_5_O)]
*M* *_r_* = 670.25
Orthorhombic, 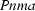

*a* = 8.1010 (1) Å
*b* = 13.5126 (2) Å
*c* = 14.0140 (2) Å
*V* = 1534.05 (4) Å^3^
*Z* = 4
Mo *K*α radiation
μ = 15.02 mm^−1^
*T* = 140 K
0.20 × 0.10 × 0.05 mm

#### Data collection

Bruker SMART APEX diffractometer
Absorption correction: multi-scan (*SADABS*; Sheldrick, 1996 ▶) *T*_min_ = 0.153, *T*_max_ = 0.521 (expected range = 0.139–0.472)
10101 measured reflections
1837 independent reflections
1752 reflections with *I* > 2σ(*I*)
*R*_int_ = 0.028

#### Refinement

*R*[*F* ^2^ > 2σ(*F* ^2^)] = 0.018
*wR*(*F* ^2^) = 0.043
*S* = 1.05
1837 reflections
91 parameters
H-atom parameters constrained
Δρ_max_ = 0.72 e Å^−3^
Δρ_min_ = −0.90 e Å^−3^

### 

Data collection: *APEX2* (Bruker, 2008 ▶); cell refinement: *SAINT* (Bruker, 2008 ▶); data reduction: *SAINT*; program(s) used to solve structure: *SHELXS97* (Sheldrick, 2008 ▶); program(s) used to refine structure: *SHELXL97* (Sheldrick, 2008 ▶); molecular graphics: *X-SEED* (Barbour, 2001 ▶); software used to prepare material for publication: *publCIF* (Westrip, 2009 ▶).

## Supplementary Material

Crystal structure: contains datablocks global, I. DOI: 10.1107/S1600536809020972/tk2471sup1.cif

Structure factors: contains datablocks I. DOI: 10.1107/S1600536809020972/tk2471Isup2.hkl

Additional supplementary materials:  crystallographic information; 3D view; checkCIF report

## Figures and Tables

**Fig. 1.**
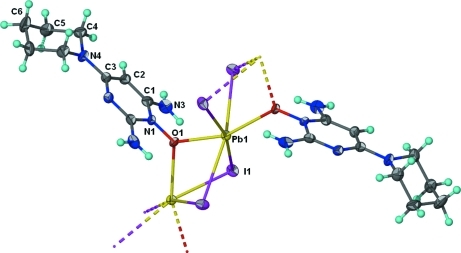
Thermal ellisoid plot (Barbour, 2001) of the lead diiodide–minoxidil adduct extended to show the coordination geometries of two lead atoms of the linear chain formed in the crystal structure. Atoms comprising the asymmetric unit are labelled, probability levels are set at 70% and H-atoms are drawn as spheres of arbitrary radius. Only one component of the disorder is shown.

## supplementary crystallographic information

### Experimental

Minoxidil [6-(1-piperidinyl)-2,4-pyrimidinediamide 3-oxide) (0.10 g, 0.5 mmol), lead(II) acetate (0.17 g, 0.5 mmol) and potassium iodide (0.16 g, 1 mmol) were placed one arm of a two-arm glass tube. Methanol was added to fill both arms. The tube was sealed and the arm containing the reactants immersed in an oil bath at 333 K while the other arm was kept at ambient temperature. After 10 days, light-brown crystals deposited in the cooler arm. These were collected, washed with acetone and ether, and finally air dried.

### Refinement

Carbon-bound H-atoms were placed in calculated positions (C–H 0.95 to 0.99 Å) and were included in the refinement in the riding model approximation, with *U*(H) set to 1.2*U*(C). The amino H-atoms were similarly treated (N–H 0.88 Å).

The minoxidil molecule is disordered about a mirror plane that is perpendicular to the pyrimidinyl ring; the mirror plane also bisects piperidinyl ring. In the aromatic ring, the nitrogen atom at the 1-position shares the same site as the carbon atom at the 5-position; this site was refined as half a nitrogen atom and half a C-H group. The short H2···H4a distance of 1.74 Å is an artifact of the disorder about a mirror plane.

### Crystal data

**Table:**

[PbI_2_(C_9_H_15_N_5_O)]	*F*(000) = 1200
*M**_r_* = 670.25	*D*_x_ = 2.902 Mg m^−^^3^
Orthorhombic, *P**n**m**a*	Mo *K*α radiation, λ = 0.71073 Å
Hall symbol: -P 2ac 2n	Cell parameters from 5726 reflections
*a* = 8.1010 (1) Å	θ = 2.9–28.3°
*b* = 13.5126 (2) Å	µ = 15.02 mm^−^^1^
*c* = 14.0140 (2) Å	*T* = 140 K
*V* = 1534.05 (4) Å^3^	Prism, yellow
*Z* = 4	0.20 × 0.10 × 0.05 mm

### Data collection

**Table:**

Bruker SMART APEX diffractometer	1837 independent reflections
Radiation source: fine-focus sealed tube	1752 reflections with *I* > 2σ(*I*)
graphite	*R*_int_ = 0.028
ω scans	θ_max_ = 27.5°, θ_min_ = 2.1°
Absorption correction: multi-scan (*SADABS*; Sheldrick, 1996)	*h* = −10→10
*T*_min_ = 0.153, *T*_max_ = 0.521	*k* = −17→17
10101 measured reflections	*l* = −18→18

### Refinement

**Table:**

Refinement on *F*^2^	Primary atom site location: structure-invariant direct methods
Least-squares matrix: full	Secondary atom site location: difference Fourier map
*R*[*F*^2^ > 2σ(*F*^2^)] = 0.018	Hydrogen site location: inferred from neighbouring sites
*wR*(*F*^2^) = 0.043	H-atom parameters constrained
*S* = 1.05	*w* = 1/[σ^2^(*F*_o_^2^) + (0.0233*P*)^2^ + 1.8105*P*] where *P* = (*F*_o_^2^ + 2*F*_c_^2^)/3
1837 reflections	(Δ/σ)_max_ = 0.001
91 parameters	Δρ_max_ = 0.72 e Å^−^^3^
0 restraints	Δρ_min_ = −0.90 e Å^−^^3^

### Fractional atomic coordinates and isotropic or equivalent isotropic displacement parameters (Å^2^)

**Table:**

	*x*	*y*	*z*	*U*_iso_*/*U*_eq_	Occ. (<1)
Pb1	0.55277 (2)	0.7500	0.769395 (12)	0.01565 (6)	
I1	0.26714 (3)	0.588041 (17)	0.831700 (16)	0.02101 (7)	
O1	0.3482 (4)	0.7500	0.6436 (2)	0.0171 (7)	
N1	0.3822 (5)	0.7500	0.5481 (3)	0.0137 (8)	
N2	0.4236 (4)	0.6615 (2)	0.4053 (2)	0.0184 (6)	0.50
N3	0.3560 (4)	0.5806 (2)	0.5498 (2)	0.0237 (7)	
H31	0.3565	0.5226	0.5213	0.028*	
H32	0.3340	0.5847	0.6112	0.028*	
N4	0.4716 (6)	0.7500	0.2638 (3)	0.0187 (9)	
C1	0.3887 (4)	0.6625 (3)	0.5000 (2)	0.0183 (7)	
C2	0.4236 (4)	0.6615 (2)	0.4053 (2)	0.0184 (6)	0.50
H2	0.4352	0.6007	0.3718	0.022*	0.50
C3	0.4417 (6)	0.7500	0.3590 (3)	0.0173 (9)	
C4	0.5041 (5)	0.6591 (3)	0.2101 (3)	0.0224 (7)	
H4A	0.4732	0.6010	0.2493	0.027*	
H4B	0.6234	0.6544	0.1956	0.027*	
C5	0.4058 (5)	0.6583 (3)	0.1176 (3)	0.0280 (9)	
H5A	0.4367	0.5992	0.0798	0.034*	
H5B	0.2867	0.6535	0.1326	0.034*	
C6	0.4363 (8)	0.7500	0.0587 (4)	0.0350 (14)	
H6A	0.5517	0.7500	0.0354	0.042*	
H6B	0.3621	0.7500	0.0026	0.042*	

### Atomic displacement parameters (Å^2^)

**Table:**

	*U*^11^	*U*^22^	*U*^33^	*U*^12^	*U*^13^	*U*^23^
Pb1	0.01521 (10)	0.01598 (10)	0.01577 (10)	0.000	−0.00072 (6)	0.000
I1	0.02066 (14)	0.01606 (12)	0.02631 (12)	−0.00058 (9)	0.00187 (9)	0.00768 (8)
O1	0.0150 (17)	0.0263 (18)	0.0099 (13)	0.000	0.0015 (12)	0.000
N1	0.0143 (19)	0.0163 (19)	0.0105 (16)	0.000	−0.0019 (14)	0.000
N2	0.0168 (16)	0.0230 (17)	0.0155 (14)	−0.0007 (13)	0.0001 (12)	−0.0035 (12)
N3	0.0270 (17)	0.0148 (15)	0.0294 (15)	−0.0038 (13)	−0.0040 (13)	−0.0053 (12)
N4	0.026 (2)	0.018 (2)	0.0118 (18)	0.000	0.0012 (16)	0.000
C1	0.0145 (16)	0.0208 (18)	0.0197 (16)	−0.0011 (14)	−0.0007 (13)	−0.0043 (13)
C2	0.0168 (16)	0.0230 (17)	0.0155 (14)	−0.0007 (13)	0.0001 (12)	−0.0035 (12)
C3	0.014 (2)	0.022 (2)	0.016 (2)	0.000	−0.0009 (18)	0.000
C4	0.0249 (19)	0.0211 (18)	0.0211 (16)	0.0032 (16)	0.0036 (15)	−0.0038 (14)
C5	0.025 (2)	0.036 (2)	0.0226 (18)	−0.0012 (17)	−0.0035 (15)	−0.0130 (16)
C6	0.039 (4)	0.051 (4)	0.014 (2)	0.000	−0.006 (2)	0.000

### Geometric parameters (Å, °)

**Table:**

Pb1—O1	2.419 (3)	N4—C3	1.356 (6)
Pb1—O1^i^	2.686 (3)	N4—C4	1.464 (4)
Pb1—I1	3.3024 (3)	N4—C4^iii^	1.464 (4)
Pb1—I1^ii^	3.1325 (3)	C3—C2^iii^	1.368 (4)
Pb1—I1^i^	3.1325 (3)	C3—N2^iii^	1.368 (4)
Pb1—I1^iii^	3.3024 (3)	C4—C5	1.522 (5)
O1—N1	1.366 (5)	C4—H4A	0.9900
N1—C1	1.363 (4)	C4—H4B	0.9900
N1—C1^iii^	1.363 (4)	C5—C6	1.509 (5)
N2—C1	1.358 (4)	C5—H5A	0.9900
N2—C3	1.368 (4)	C5—H5B	0.9900
N3—C1	1.335 (4)	C6—C5^iii^	1.509 (5)
N3—H31	0.8800	C6—H6A	0.9900
N3—H32	0.8800	C6—H6B	0.9900
			
O1—Pb1—O1^i^	160.22 (9)	C4—N4—C4^iii^	114.1 (4)
O1—Pb1—I1^i^	92.88 (5)	N3—C1—N2	123.0 (3)
O1^i^—Pb1—I1^i^	73.21 (5)	N3—C1—N1	116.9 (3)
O1—Pb1—I1^ii^	92.88 (5)	N2—C1—N1	120.1 (3)
O1^i^—Pb1—I1^ii^	73.21 (5)	N4—C3—N2	119.0 (2)
I1^i^—Pb1—I1^ii^	88.636 (10)	N4—C3—C2^iii^	119.0 (2)
O1—Pb1—I1	73.30 (5)	N2—C3—C2^iii^	121.9 (4)
O1^i^—Pb1—I1	120.29 (4)	N4—C3—N2^iii^	119.0 (2)
I1^i^—Pb1—I1	92.581 (7)	N2—C3—N2^iii^	121.9 (4)
I1^ii^—Pb1—I1	166.169 (8)	C2^iii^—C3—N2^iii^	0.0 (3)
O1—Pb1—I1^iii^	73.30 (5)	N4—C4—C5	110.5 (3)
O1^i^—Pb1—I1^iii^	120.29 (4)	N4—C4—H4A	109.6
I1^i^—Pb1—I1^iii^	166.169 (8)	C5—C4—H4A	109.6
I1^ii^—Pb1—I1^iii^	92.581 (7)	N4—C4—H4B	109.6
I1—Pb1—I1^iii^	83.011 (9)	C5—C4—H4B	109.6
Pb1^iv^—I1—Pb1	78.806 (5)	H4A—C4—H4B	108.1
N1—O1—Pb1	125.2 (3)	C6—C5—C4	112.0 (4)
N1—O1—Pb1^iv^	128.6 (3)	C6—C5—H5A	109.2
Pb1—O1—Pb1^iv^	106.25 (11)	C4—C5—H5A	109.2
O1—N1—C1	119.5 (2)	C6—C5—H5B	109.2
O1—N1—C1^iii^	119.5 (2)	C4—C5—H5B	109.2
C1—N1—C1^iii^	120.5 (4)	H5A—C5—H5B	107.9
C1—N2—C3	118.5 (3)	C5^iii^—C6—C5	110.4 (4)
C1—N3—H31	120.0	C5^iii^—C6—H6A	109.6
C1—N3—H32	120.0	C5—C6—H6A	109.6
H31—N3—H32	120.0	C5^iii^—C6—H6B	109.6
C3—N4—C4	122.51 (19)	C5—C6—H6B	109.6
C3—N4—C4^iii^	122.51 (19)	H6A—C6—H6B	108.1
			
O1—Pb1—I1—Pb1^iv^	−35.49 (5)	C3—N2—C1—N3	174.6 (4)
O1^i^—Pb1—I1—Pb1^iv^	160.24 (6)	C3—N2—C1—N1	−3.9 (5)
I1^i^—Pb1—I1—Pb1^iv^	−127.726 (10)	O1—N1—C1—N3	1.9 (5)
I1^ii^—Pb1—I1—Pb1^iv^	−32.94 (2)	C1^iii^—N1—C1—N3	−169.9 (3)
I1^iii^—Pb1—I1—Pb1^iv^	39.115 (7)	O1—N1—C1—N2	−179.5 (3)
O1^i^—Pb1—O1—N1	0.000 (2)	C1^iii^—N1—C1—N2	8.7 (7)
I1^i^—Pb1—O1—N1	−44.389 (6)	C4—N4—C3—N2	−7.1 (7)
I1^ii^—Pb1—O1—N1	44.389 (6)	C4^iii^—N4—C3—N2	−175.6 (4)
I1—Pb1—O1—N1	−136.222 (16)	C4—N4—C3—C2^iii^	175.6 (4)
I1^iii^—Pb1—O1—N1	136.222 (17)	C4^iii^—N4—C3—C2^iii^	7.1 (7)
O1^i^—Pb1—O1—Pb1^iv^	180.0	C4—N4—C3—N2^iii^	175.6 (4)
I1^i^—Pb1—O1—Pb1^iv^	135.611 (6)	C4^iii^—N4—C3—N2^iii^	7.1 (7)
I1^ii^—Pb1—O1—Pb1^iv^	−135.611 (6)	C1—N2—C3—N4	−178.0 (4)
I1—Pb1—O1—Pb1^iv^	43.778 (16)	C1—N2—C3—C2^iii^	−0.8 (7)
I1^iii^—Pb1—O1—Pb1^iv^	−43.778 (16)	C1—N2—C3—N2^iii^	−0.8 (7)
Pb1—O1—N1—C1	94.1 (3)	C3—N4—C4—C5	134.8 (5)
Pb1^iv^—O1—N1—C1	−85.9 (3)	C4^iii^—N4—C4—C5	−55.8 (6)
Pb1—O1—N1—C1^iii^	−94.1 (3)	N4—C4—C5—C6	54.1 (5)
Pb1^iv^—O1—N1—C1^iii^	85.9 (3)	C4—C5—C6—C5^iii^	−53.5 (6)

Symmetry codes: (i) *x*+1/2, *y*, −*z*+3/2; (ii) *x*+1/2, −*y*+3/2, −*z*+3/2; (iii) *x*, −*y*+3/2, *z*; (iv) *x*−1/2, *y*, −*z*+3/2.
